# Telomere attrition and restoration in the normal teleost *Oryzias latipes* are linked to growth rate and telomerase activity at each life stage

**DOI:** 10.18632/aging.100873

**Published:** 2016-01-20

**Authors:** Hitoshi Hatakeyama, Hiromi Yamazaki, Ken-Ichi Nakamura, Naotaka Izumiyama-Shimomura, Junko Aida, Hiroetsu Suzuki, Shuichi Tsuchida, Masaaki Matsuura, Kaiyo Takubo, Naoshi Ishikawa

**Affiliations:** ^1^ Department of Comprehensive Education in Veterinary Medicine, Nippon Veterinary and Life Science University, Tokyo 180-8602, Japan; ^2^ Research Team for Geriatric Diseases, Tokyo Metropolitan Institute of Gerontology, Tokyo 173-0015, Japan; ^3^ Department of Basic Veterinary Medicine, Nippon Veterinary and Life Science University, Tokyo 180-8602, Japan; ^4^ Bioinformatics Group, Genome Center, and Division of Cancer Genomics, Cancer Institute, Japanese Foundation for Cancer Research, Tokyo 135-8550, Japan

**Keywords:** telomere, telomerase, aging, growth, adolescence, medaka

## Abstract

Telomere shortening occurs when cells divide, both *in vitro* and *in vivo*. On the other hand, telomerase is able to maintain telomere length in cells by adding TTAGGG repeats to the ends of telomeres. However, the interrelationships existing among telomere length, telomerase activity and growth in vertebrates remain to be clarified. In the present study we measured telomere length (terminal restriction fragment length), telomerase activity and body growth of *Oryzias latipes* from the embryo stage until senescence. During the rapid growth stage (age 0–7 months), telomeres shortened in parallel with decreasing telomerase activity. Then, during adolescence (age 7 months – 1 year), telomeres lengthened quickly as growth slowed and telomerase activity increased. In the adult stage (age 1–4 years) characterized by little growth, telomerase activity decreased gradually and telomeres shortened. Our data indicate that telomere attrition and restoration are linked to growth and telomerase activity, and suggest that critical loss of telomere homeostasis is associated with mortality in this animal.

## INTRODUCTION

Telomeres, located at the ends of chromosomes, are composed of a few dozen base pairs (bp) to several hundred kilobase pairs (kbp) of duplex GT-rich 5′-TTAGGG-3′ repeats in most metazoans [1]. Because of inefficient DNA replication resulting from finite end replication [2] and T loop loss [3], telomeres shorten with age during the life of an individual animal. When telomere length falls below a critical threshold level, replicative senescence or cell death ensues [4].

Progressive telomere attrition has been linked to both normal aging and various degenerative diseases in a wide range of studies mainly involving humans and mice [5]. By contrast, telomere extension has been shown to have a restorative effect [6]. In most metazoans, the enzyme telomerase, a cellular reverse transcriptase, promotes telomeric repair and reduces telomere erosion by adding conserved TTAGGG repeats to chromosome ends [7].

Studies of telomere length in humans have focused on immortalized cells [8] and induced pluripotent stem (iPS) cells [9] cultured in the presence of telomerase *in vitro*, or on aging and longevity [10] in the absence of telomerase activity *in vivo*. Consequently, no comprehensive overview of telomere shortening in relation to cell division and the life cycle has emerged [11]. Some studies have suggested that telomere attrition is rapid during early life when growth is still occurring [12, 13], and then becomes more gradual during adult life when cell turnover becomes steady [14]. A few studies of humans have suggested that telomere length remains stable between childhood and adolescence [12], and that – in accordance with the telomere hypothesis – telomere shortening in normal somatic cells in the absence of telomerase acts as a mitotic clock for replicative senescence [15]. Recently, there has been an upsurge of interest in telomere dynamics in healthy organisms [16]. Telomere research has also entered a new stage that encompasses areas such as lifespan prediction [17], age estimation in ecology [18], factors affecting telomere attrition [19, 20, 21, 22, 23] and telomere homeostasis through covalent attachment of a small ubiquitin-like modifier (SUMO) to target proteins (SUMOylation) [24, 25]. However, much remains to be clarified regarding the significance of telomere dynamics in a cross-species context, and studies of the relationships between patterns of telomere loss and restoration, biodiversity, and the life history of organisms remain in their infancy [16]. In addition, opinions on telomere measurement have been divided, and recent papers have called for standardization of methodologies used for telomere measurement and analysis [26, 27, 28]. Researchers have therefore begun to compare and analyze representative methodologies for telomere measurement [29].

Fish are one of the five classes of vertebrates, and continue to grow throughout life [30]. Teleost fish have been used extensively for studies on growth [30], aging [31] and telomere biology [32], especially small laboratory fish such as the zebrafish [33] and medaka [34]. The zebrafish, *Danio rerio*, is an animal that has been widely employed as a model in studies of growth and aging [33]. However, unlike most vertebrates, telomerase activity in this species persists throughout life, and the telomere and telomerase dynamics *in vivo* do not result in telomere attrition with age [35]. The medaka, *Oryzias latipes*, is also a widely used animal model [34] for which the draft genome sequence is now available [36], and furthermore, like most vertebrates, shows telomere attrition with age despite possessing telomerase activity [37, 38]. The channel catfish, *Ictalurus punctatus*, a phenomenon that appears to conflict with the established telomere hypothesis *in vitro* [15], as immortal leukocytes in this species appear to show a decrease in telomere length until a critical limit is reached, even in the presence of telomerase activity [39].

Previously, we have reported that data on telomere and telomerase dynamics during the lifetime of the medaka are incomplete, due to an absence of data for the adolescent stage (age 7 months – 1 year) and differences in the method of sampling in the growth and senescence stages, when sampling was conducted for the whole body and systemic organs, respectively [38]. Furthermore, we assessed the telomere length and body size of the medaka using only linear regression analysis, and compared the data for telomerase activity with that for a human cancer cell line. In the present study, we measured in detail both telomere (terminal restriction fragment; TRF) length and telomerase activity in samples extracted from the whole body, body length and weight of the medaka from the embryo stage until senescence, and conducted model selection exercises based on information theoretic criteria [40, 41]. We found that telomeres do not shorten linearly with age, but in accordance with the growth rate and level of telomerase activity at each life stage. Our findings challenge the long-standing view of telomere dynamics, and provide a unique example of the interrelationships existing among telomere length, telomerase, growth and aging. Moreover, we discuss the issue of analytical methodologies for telomere measurement.

## RESULTS

### Medaka growth, body size and life span

**Figure 1:**
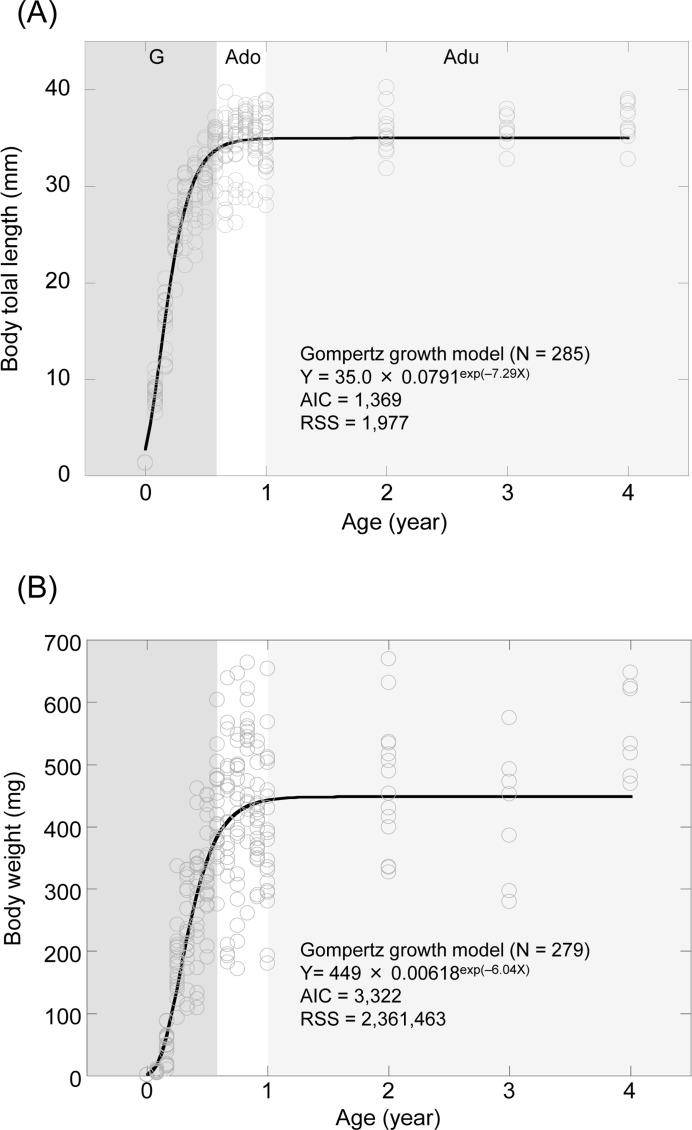

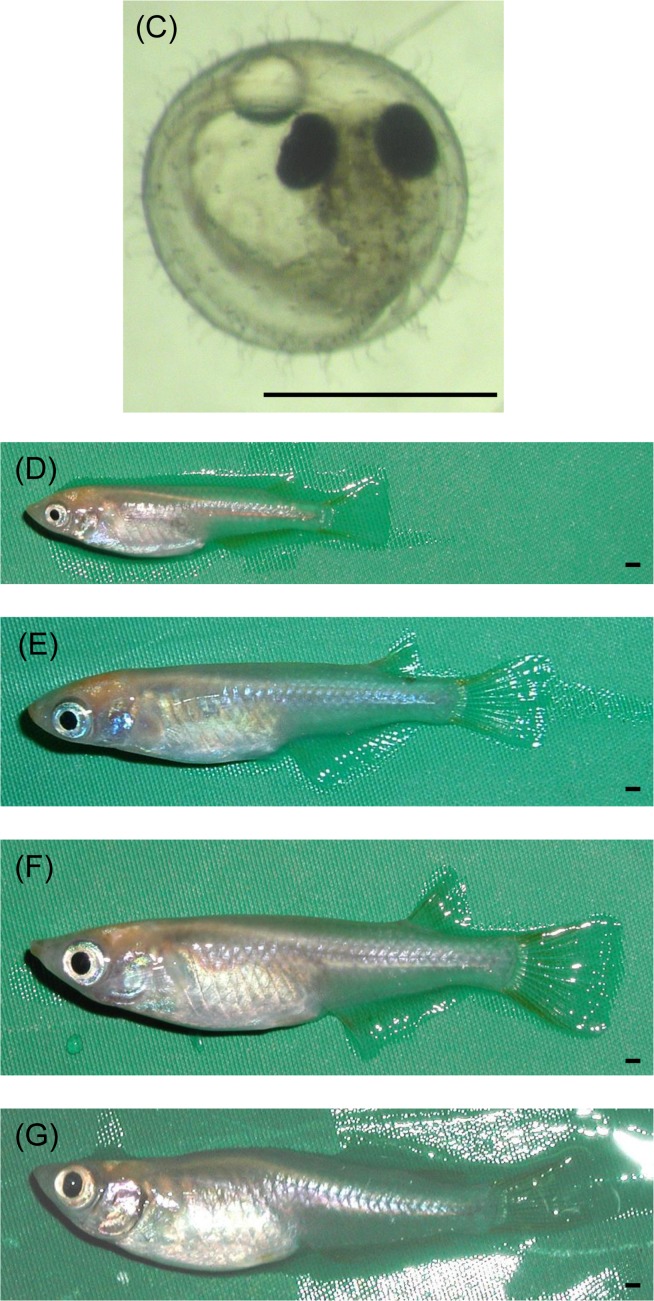

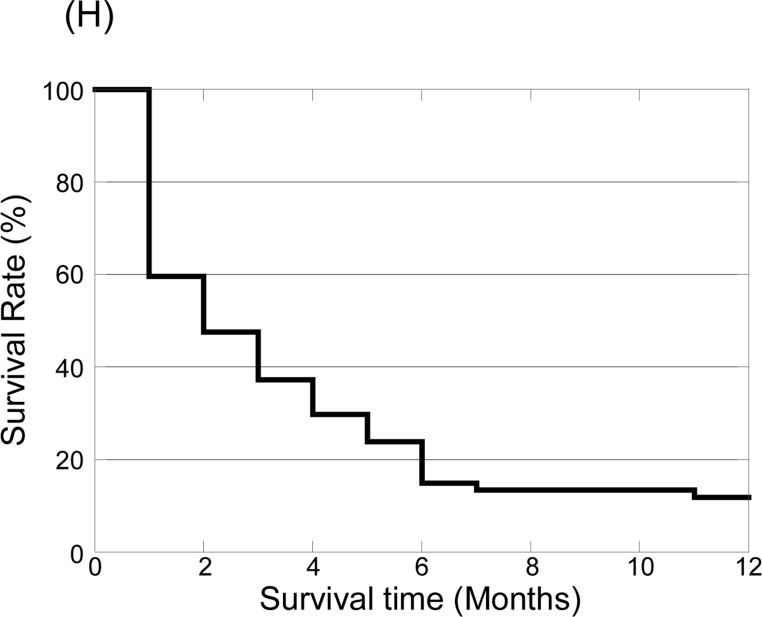
Characteristics of the medaka (*Oryzias latipes*) at different ages (**A, B**) Scatterplots of the dynamics and fitted regression models. (**A**) Total body length. (**B**) Body weight. G (dark gray shading), growth stage; Ado (clear), adolescent stage; Adu (light gray shading), adult stage. (**C-G**) Changes in external morphology of the medaka. (**C**) Embryo body at 10 days of age (hatching stage). (**D**) Female at 3 months of age at the growth stage. (**E**) Female at 11 months of age at the adolescent stage. (**F**) Female at 2 years of age at the adult stage. (**G**) Female at 4 years of age at the adult stage. Scale bar = 1 mm. (**H**) Kaplan-Meier survival estimate for medaka from hatching to 1 year of age (*N* = 67).

To evaluate the body size dynamics (Table 1), we applied the data for body length and weight to four models; linear regression, exponential regression, asymptotic regression and the Gompertz growth model. Model selection was accomplished by selecting from among the candidate models the one that minimized Akaike Information Criterion (AIC) and Residual Sum of Squares (RSS). We judged the Gompertz growth model to be the most appropriate model for assessing both body length (Figure 1A and Table 2) and weight (Figure 1B and Table 2) from the AIC, and also from the RSS as a reference (Table 2). The scatter plots of medaka body length (Figure 1A) and weight (Figure 1B) indicated rapid growth in the maximal growth stage (before 7 months), slower growth in the adolescent stage (7 months to 1 year) and hardly any growth in the adult stage (after 1 year). The degree of growth was also reflected in the external morphology (Figure 1C-G), although there were differences in terms of the hallmarks of aging in the adult stage (Figre 1F, G). The life span data from hatching to 1 year were derived from part of a population bred in 2009 (Figure 1H). The survival curve of medaka showed a constant decline during the growth stage (from hatching to 7 months) and was almost flat during the adolescent stage (from 7 months to 1 year).

**Table 1:** Age demographic characteristics in medaka

	Body length [mm]	Body weight [mg]	TRF [kbp], median by Telometric	TRF [kbp], mean by ImageJ	TRAP [RTA]
embryo 10 dy	1.4±0.1(6)*	1.3±0.1(6)	13.1±1.7(6)**	8.9±0.8(6)**	1.23±0.17(4)
1 mo	8.7±1.0(23)	6.7±1.7(23)	13.9±1.1(19)	10.9±1.0(19)	1.50±0.12(4)
2 mo	15.9±2.8(19)	46.8±23.2(19)	12.7±1.3(15)	9.6±1.8(15)	1.25±0.16(4)
3 mo	25.2±2.2(20)	181.0±51.2(20)	13.6±1.5(16)	11.5±1.5(16)	1.26±0.14(4)
4 mo	28.2±2.6(20)	222.7±84.0(20)	13.2±1.5(16)	10.0±1.7(16)	1.20±0.21(4)
5 mo	29.8±3.2(21)	283.5±94.5(21)	12.4±1.3(17)	9.0±1.3(17)	1.19±0.15(4)
6 mo	31.2±2.3(20)	329.2±72.0(20)	11.4±1.1(16)	7.7±1.0(16)	1.22±0.16(4)
7 mo	34.8±1.9(21)	430.3±75.2(21)	11.1±1.1(17)	7.3±0.9(17)	1.32±0.08(4)
8 mo	33.5±3.6(21)	381.1±135.2(21)	12.4±1.3(17)	9.5±1.4(17)	1.21±0.20(4)
9 mo	34.2±3.2(20)	411.0±123.1(20)	12.7±1.5(18)	8.4±1.6(18)	1.19±0.18(4)
10 mo	35.4±2.4(20)	447.9±108.2(20)	12.6±1.5(16)	9.4±2.1(16)	1.27±0.35(4)
11 mo	35.0±2.2(20)	404.0±68.7(20)	12.6±1.1(16)	11.2±2.3(16)	1.53±0.18(4)
1 yr	34.5±2.2(20)	390.5±121.4(20)	14.7±1.2(16)	13.1±1.4(16)	1.69±0.19(4)
2 yr	36.0±2.1(14)	479.3±105.4(14)	12.5±2.0(13)	9.3±2.1(13)	1.48±0.26(4)
3 yr	35.9±1.8(10)	421.7±112.6(7)	12.7±1.0(11)	9.4±1.6(11)	1.20±0.06(4)
4 yr	36.7±2.0(10)	556.3±72.4(7)	11.5±1.2(10)	7.7±1.1(10)	1.19±0.07(4)

The mean values are given with one standard deviation (S.D.).

Numbers examined are indicated in parenthesis.

* Embryo 10 dy measured in diameter.

** Each of the embryonic samples consisted of 10 pooled individuals.

**Table 2:** Model selection for body growth dynamics in medaka

Model	Formula	AIC	RSS
*Body length*			
Linear regression	Y= 5.49X + 24.6	2,004	18,515
Exponential regression	Y= 33.3 X^0.219^	1,764	7,966
Asymptotic regression	Y= −37.7 × 0.0125^X^ + 36.0	1,377	2,034
Gompertz growth	Y= 35.0 × 0.0791^exp(−7.29X)^	1,369	1,977
*Body weight*			
Linear regression	Y= 130 X + 212	3,588	6,157,493
Exponential regression	Y= 390 X^0.384^	3,471	4,046,615
Asymptotic regression	Y= −594 × 0.0718^X^ + 489	3,338	2,490,122
Gompertz growth	Y= 449 × 0.00618^exp(−6.04X)^	3,322	2,361,463

**Table 3:** Model selection for telomere and telomerase dynamics in medaka

Model	Formula	AIC_C1_	RSS
*Telomere dynamics by Telometric*			
Linear regression	Y= −0.262 X + 13.0	465.9	602.2
Nonparametric regression (LOWESS, smoothing parameter value 0.165)	–	403.2	410.2
*Telomere dynamics by ImageJ*			
Linear regression	Y= −0.327 X + 9.91	603.9	1072.3
Nonparametric regression (LOWESS, smoothing parameter value 0.165)	–	470.2	542.9
*Telomerase dynamics*			
Linear regression	Y= −0.010 X + 1.318	−126.0	2.976
Nonparametric regression (LOWESS, smoothing parameter value 0.352)	–	−144.3	1.864

### Medaka telomere dynamics

**Figure 2:**
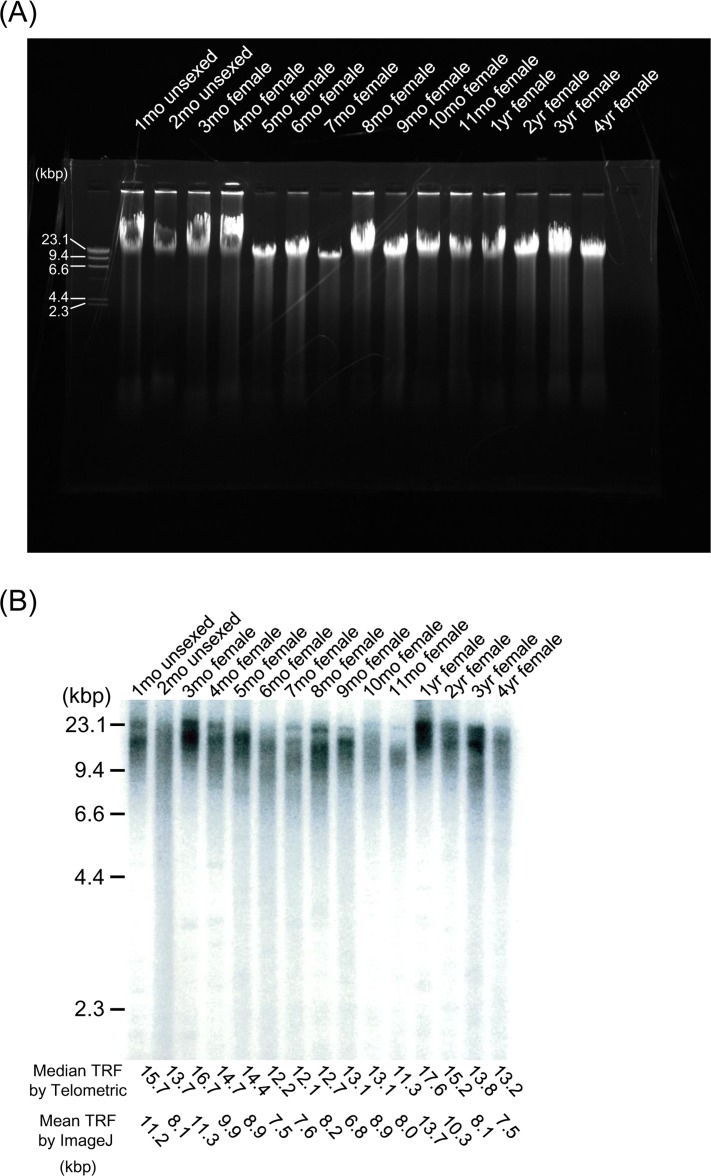

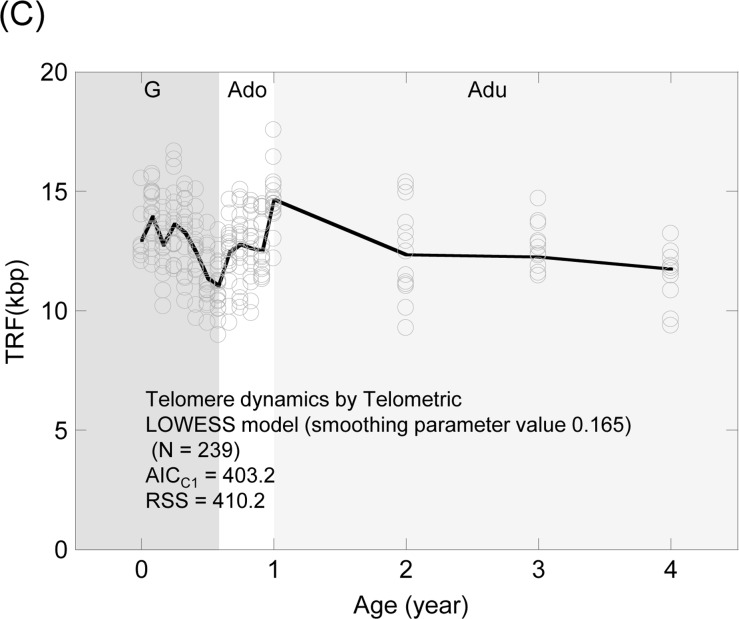
TRF length from embryo to extreme old age in the medaka (**A**) Representative gel electrophoresis analysis using genomic DNA from the whole body at different ages. DNA samples were resolved on a 0.8% (wt/vol) agarose gel at 100V for 30 min. All lanes contain DNA larger than the 23.1-kbp marker, and appear as single compact crowns that have migrated in parallel as an undegraded intact sample. (**B**) Representative Southern blot analysis using genomic DNA from the whole body at different ages. The TRF of telomeric DNA yielded wide smears in all lanes. TRF values (median values determined using Telometric version 1.2 and mean values determined using ImageJ version 1.39) (kbp) are listed at the bottom. (**C**) Scatterplots of telomere dynamics obtained using Telometric and the fitted regression model. G (dark gray shading), growth stage; Ado (clear), adolescent stage; Adu (light gray shading), adult stage.

Prior to restriction digestion, genomic DNA from all samples was tested for autolytic changes by gel electrophoresis (Figure 2A). All samples contained DNA larger than the 23.1kbp, and appeared as single compact crowns that have migrated in parallel as an undegraded intact sample. Telomere (terminal restriction fragment; TRF) length showed considerable heterogeneity among age-matched samples from the embryo stage to extreme old age (Figure 2B, C, Table 1 and Supplemental Figure S1A). We took at least 6 samples (more than 10, except for embryos) at each age and used the mean values as representatives. We applied linear regression models, which are widely used in studies of telomere dynamics, and the Locally Weighted Scatterplot Smoothing (LOWESS) model, a non-parametric regression model, to analyze the median TRF data obtained by Telometric, and found that both models were suitable for statistical analysis. When we chose an appropriate smoothing parameter value to minimize AIC_C1_ which is the bias-corrected AIC, the LOWESS model was judged to be a better fitted model than the linear regression model from AIC_C1_ (Figure 2C and Table 3), and also from RSS as a reference (Table 3). Furthermore, we repeated the same analysis of the mean TRF data obtained by ImageJ, and selected the same model as that used in previous analysis employing Telometric (Supplemental Figure S1A and Table 3). The LOWESS model demonstrated that the TRF length in medaka obtained by Telemetric and ImageJ decreased rapidly with oscillation during the growth stage, then increased quickly during the adolescent stage and became maximal at the fully mature stage (1 year of age), before again decreasing gradually in the adult stage. In the adult stage, the rate of TRF length shortening between the ages of 1 and 2 years was higher than that after 2 years of age (Figure 2C, Table 3 and Supplemental Figure S1A). Our mean TRF lengths assessed using ImageJ were shorter than those obtained using Telometric (median TRF length) (Table 1), but this difference had virtually no qualitative effect on the pattern of dynamics (Supplemental Figure S1B).

### Medaka telomerase dynamics

**Figure 3:**
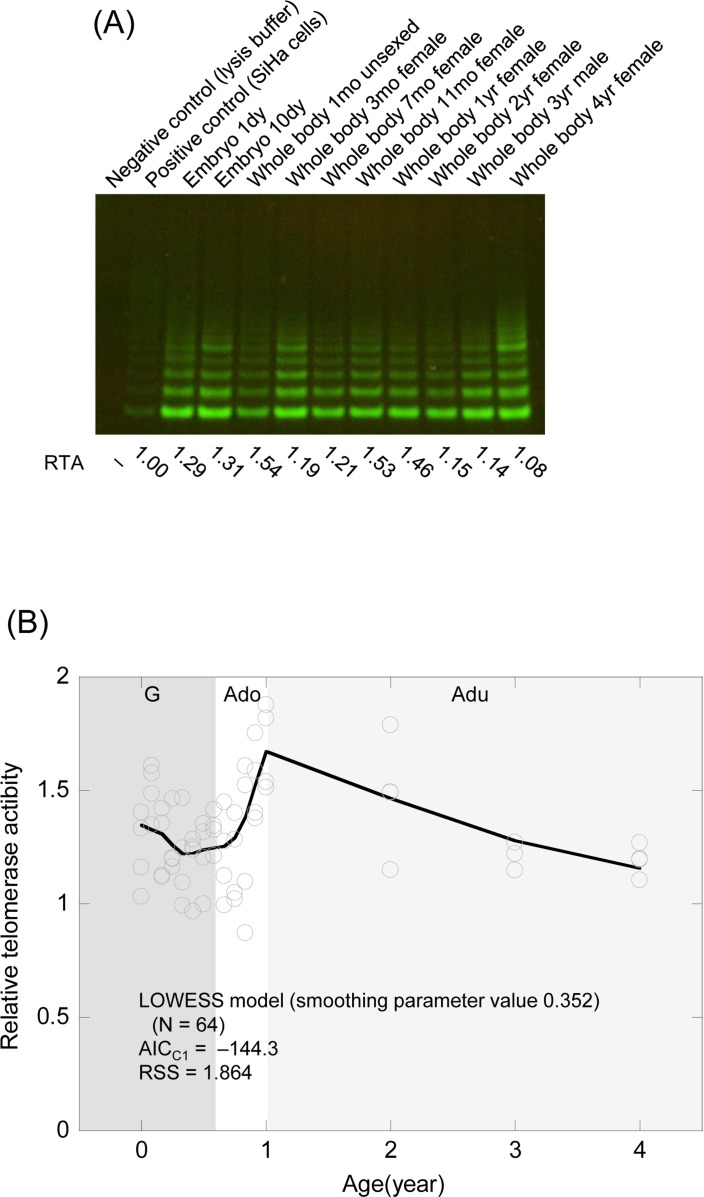
Telomerase activity from the embryo stage to senescence in the medaka (**A**) Representative TRAP assay for the embryo stage and the whole body of the adult. RTA values (Related Telomerase Activity; the activity relative to that in SiHa cells as a positive control) are listed at the bottom. (**B**) Scatter plots of telomerase dynamics and the fitted regression model. G (dark gray shading), growth stage; Ado (clear), adolescent stage; Adu (light gray shading), adult stage.

We assessed telomerase activity by the Telomeric Repeat Amplification Protocol (TRAP) assay using samples taken over the whole life cycle from the embryo stage to senescence (Table 1). Since telomerase activity showed considerable heterogeneity similar to that of telomere (TRF) length among age-matched samples, we carried out assays in quadruplicate and used the mean values as representatives. These values were not more than twice those for the human cancer cell line (SiHa cell) used as a positive control (Figure 3A, B). Although the telomerase activity data were fitted a linear regression model and the LOWESS model chosen as the smoothing parameter to minimize AIC_C1_, the LOWESS model was again judged to be a more appropriate model than the linear regression model from AIC_C1_ (Figure 3B and Table 3), using RSS as a reference (Table 3). The LOWESS model indicated that medaka telomerase activity fell gradually during the growth stage, increased gradually from the late growth phase and then increased rapidly during the adolescent stage, before gradually lessening again in the adult stage. Thus, telomerase dynamics in the medaka paralleled telomere dynamics.

### Relationship between telomere length and telomerase activity

**Figure 4:**
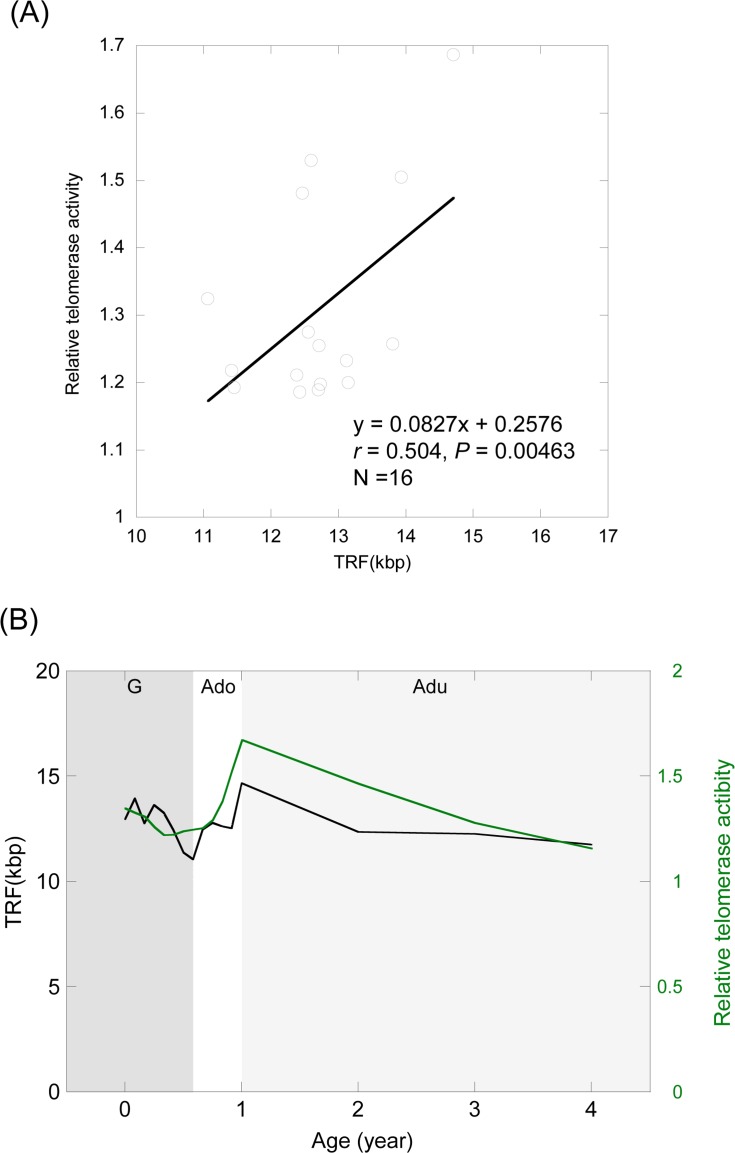
Relationship between telomere length and telomerase activity (**A**) Correlation between the average telomere length and telomerase activity at the same age. (**B**) Comparison of the regression curve between telomere dynamics and telomerase dynamics. Black curve, TRF length; Green curve, telomerase activity. G (dark gray shading), growth stage; Ado (clear), adolescent stage; Adu (light gray shading), adult stage.

To clarify the relationship between TRF length and telomerase activity, we examined the correlation between the two parameters in individuals of the same age (*r* = 0.504, *P* = 0.00463; Pearson's correlation) (Figure 4A). We also compared the regression curve of telomere dynamics against telomerase dynamics (Figure 4B). The level of telomerase activity was correlated with telomere length, although changes in telomere dynamics slightly lagged behind those in telomerase dynamics (Figure 4B).

## DISCUSSION

In our previous report [38] we speculated that telomeres might elongate during the adolescent period because telomere lengths in the whole body at age 6 months and in systemic organs at 1 year were about 10 and 15 kbp, respectively, and intra-individual synchrony of telomere length exists in somatic tissues, not only in medaka [38], but also in human [10, 42]. To obtain our present results, we added samples that had been lacking in previous experiments and applied new approaches for statistical analysis. We reassessed the changes in telomere dynamics in the medaka by dividing its life history into three growth stages. First, in the period of rapid growth (before 7 months), telomeres shortened promptly, while exhibiting some fluctuations when growth was accelerated, being correlated with a decrease of telomerase activity. Then, in the adolescent stage (between 7 months and 1 year), telomere length increased quickly with some fluctuations, and became maximal at the fully mature stage, in parallel with slow growth and increasing telomerase activity. Finally, in the adult stage (after 1 year), telomere length became gradually reduced in parallel with minimal growth and low telomerase activity. These findings clearly demonstrate that telomeres do not simply shorten throughout life but increase and decrease in length several times during the various life stages of the medaka, reflecting the rate of growth and level of telomerase activity at each stage. Our results suggest that telomere shortening is accompanied by rapid growth or gentle cell turnover, and that telomere elongation requires a certain level of telomerase activity. The rate of telomere shortening in younger adults was higher than that in older adults. Our results suggest that the disparity of telomere shortening makes a difference depending on cell turnover during that period [12, 14]. Telomere length in fish might appear to decrease with age *in vitro* [39] because telomerase activity might not reach a level sufficient for sustaining telomere homeostasis. The dynamics of telomeres and telomerase in a vertebrate *in vitro* differ from those *in vivo*, and further comparative studies are required to obtain a comprehensive consensus so that the mechanism of telomere homeostasis *in vitro* [24, 25] can be applied to regenerative medicine. Although telomerase activity in this fish species is about the same or higher than that in the human cancer cell line we employed (SiHa cells) throughout life, telomeres do not simply shorten in the period of rapid growth and elongate during adolescence but change with some fluctuations in both periods. This phenomenon suggests that telomeres undergo regulation to maintain their length within a certain range to prevent rapid shortening and elongation.

The stage of telomere reduction and telomerase depression observed in this study appears to correlate with the stage of high mortality observed in Figure 1H and previous studies [43, 44], and parallels the specific stage of growth. By contrast, the stage of telomere elongation and elevated telomerase activity in the present study appears to correspond to the stage of low mortality confirmed in Figure 1H and previous studies [38, 43, 44]. Therefore, we infer that telomere length is closely associated with mortality.

Over the last few years, we have addressed new methodologies for analysis of telomere length. We initially implemented a method used previously in telomere research [45] and estimated the mean telomere length from the peak position on the autoradiogram profile [46]. Subsequently, we used the Telometric software package to perform reproducible measurements of telomere length [47] and adopted the median value as representative of telomere length [38, 48]. We additionally confirmed the correlation between the median values obtained using Telometric and the peak values [48]. However, the Telometric program we used [38, 48] has been criticized for yielding biased results [26, 28, 49]. The underlying algorithm of Telometric interpolates data at high molecular weights, thus exaggerating telomere length in comparison with software such as ImageJ [26]. Olsson el al. [29] compared the telomere lengths obtained using Telometric and ImageJ. ImageJ yielded shorter average telomere length values, but this difference had virtually no qualitative effect on the overall pattern. We also compared the telomere lengths estimated by Telometric and ImageJ, and obtained results similar to those of Olsson et al. [29]. Haussmann et al. [26] argued that the greater the telomere length estimated using Telometric, the greater the difference between it and the value obtained using ImageJ. Conversely, as illustrated in Supplemental Figure S1B, the greater the telomere length estimated using Telometric, the difference between it and that obtained using ImageJ was, in fact, less. Although the reason for these conflicting results is uncertain, we think that it might be related to the fact that the mean value as a representative of telomere length obtained using ImageJ was adopted in spite of the non-normally distributed autoradiogram profile. The uncorrected technique based on the ImageJ image analysis program mathematically underestimates the telomere length because migration of DNA in agarose gels follows a log-linear distribution [50]. Therefore, we consider that Telometric allows conversion to a linear molecular weight profile through log linear interpolation of molecular weight to the distance travelled, because measurement of telomere length is not based on estimation of molecular weight from the distance travelled by a band, but calculates a representative value such as a mean and a median from a region in which a smear has moved logarithmically [47].

Telomere dynamics *in vitro* have been frequently estimated in terms of population doubling (PD) units [8, 11]. PD is applied as a measure of not only growth but also time. On the other hand, telomere dynamics *in vivo* have been estimated in units of time [10, 11, 12, 13, 14, 17, 18, 37, 38]. Time cannot be applied as a measure of growth like PD because vertebrates differ in their growth rates at various life stages. Therefore, the profile of growth should be estimated in terms of telomere dynamics, as telomere shortening is dependent on cell division, i.e. proliferation [15]. Similarly, the profile of telomerase activity should also be estimated in terms of telomere dynamics, since telomere elongation is dependent on telomerase activity [51]. Telomere dynamics *in vivo* need to be understood in terms of not only time but also growth, in order to clarify why telomere attrition and restoration occur. However, current knowledge of telomere dynamics, together with growth and telomerase activity profiles, is very limited [38]. In the present study, therefore, we addressed the relationship between telomere and telomerase dynamics and body growth.

Several studies have investigated the growth of fish, including the medaka [30, 38, 52]. However, when we re-evaluated the growth of the medaka by model selection [40] in the present study, we found that growth did not continue throughout life, contrary to previous knowledge. As is evident from Figure 1A and B, after adolescence the medaka measured less than 4.0 cm in length and weighed less than 700 mg at maximum. Fish exhibit phenotypic (adaptive) plasticity [53] in being able to resume growth if transferred to a better environment, but – as is the case for humans [54] – it is inconceivable that the medaka would continue to grow throughout its lifetime even if circumstances permit.

When conducting the present study, we considered that a thorough understanding of telomere and telomerase dynamics during the entire lifetime of a typical simple vertebrate would be valuable. However, our findings are limited by the scope of our study, in which the sample size was small, sampling was conducted at only a few age points, sampling was biased at specific time points, and intervals between sampling were insufficient. In vertebrates, telomerase dynamics are more difficult to understand because telomerase activity cannot be detected in normal human somatic tissues [51] and information on telomerase dynamics in normal somatic tissues of other animals is currently very limited [16]. However, we did succeed in revealing telomere and telomerase dynamics during the entire lifetime of the medaka, especially the adolescent stage for which details have been reported only infrequently. Our finding that telomere length increased due to higher telomerase activity in adolescence was surprising, although this can perhaps be viewed as compensating for early drastic telomere attrition at this stage. This higher telomerase activity in adolescence reflected previous findings reported for the same species [37]. Accordingly, we propose that telomere and telomerase dynamics at the adolescent stage need to be re-evaluated in humans because rapid telomere shortening occurs at a time of growth, and humans continue to grow until adolescence; furthermore a growth spurt is observed during adolescence [54]. If telomere length is not restored after a period of rapid attrition, and given the fact that progressive telomere attrition is linked to various degenerative diseases [5], individual organisms might evolve a survival strategy to avoid telomere shortening by limiting their body growth.

In this study, we were able to clarify telomere and telomerase dynamics at various stages of the medaka life cycle. As is evident from Figure 4A and B, telomere dynamics are correlated with telomerase dynamics. In view of this apparent correlation, our present results suggest that telomerase may be responsible for precise control of telomere length, or more specifically, the fact that telomere dynamics lag slightly behind telomerase dynamics may reflect the fact that telomerase helps to maintain telomere length. It is possible that the medaka may grow to a species-specific stage size as a result of telomere shortening, which acts as an internal “counter” of the number of cell divisions [23], and telomere length may be restored in the adolescent stage, ahead of the gradual shortening that will occur as a result of lower cell turnover in the adult stage. Although some previous studies have investigated how the size of an organism is determined *in vitro* [55, 56], *in vivo* data are very limited. Our present data suggest that regulation of growth and/or cell turnover by telomeres and telomerase are closely involved in body size control *in vivo*.

In conclusion, our present results show that telomeres in the medaka do not shorten linearly with age, and change according to the growth rate and level of telomerase activity at each life stage. Our data suggest that the link between telomeres and telomerase controls overall life history, including aspects such as growth and aging. We have also shown that telomere length is restored for a short period during the adolescent stage in the medaka. Further studies of other vertebrate species will be required in order to shed further light on telomere and telomerase dynamics at the adolescent stage. Although studies of telomere and telomerase dynamics have been limited because of their labor-intensive nature [57], such studies are important in order to obtain fundamental data for understanding the mechanisms of growth and aging.

## MATERIALS AND METHODS

### Fish (*Oryzias latipes*)

We used a population of outbred medaka maintained for more than 10 years at Nippon Veterinary and Life Science University, from which the life span data have been characterized and reported previously [38]. All the fish analyzed in the present study had been bred in 2009, originating from paired adult fish (*N* = 24; 11 males and 13 females in total) taken from the population at our laboratory. One male and three female fish died during the breeding period. The paired fish were kept in the breeding tank, which were 35-litre aquaria (30 × 45 × 30 cm), maintained at 30 ± 1(C under a 15 h: 9 h light: dark photoperiod, with dawn at 08:00 hours to encourage breeding. When we found female fish with eggs attached to their abdomens, we caught them and transferred the eggs to the breeding tanks contained aerated well water, gravel to a depth of 1 cm, and an aquarium heater. This series of experiments was started from about 1,800 eggs collected as described above.

After hatching, the juvenile medaka were reared in mixed-sex stock tanks of the same size as the breeding tank, which were maintained at 25 ± 1(C under a 12 h: 12 h light:dark photoperiod with dawn at 08:00 hours. According to their growth, the fish were transferred to stock tanks each contained a maximum of 100 individuals aged less than 7 months, and 50 individuals aged over 7 months, respectively, under the same conditions as those for the breeding tank except the temperature, light and population density conditions. The medaka were fed Tetra Min flake food (Tetra, Germany) twice a day until satiation. The eggs were then transferred to another tank. Embryonic bodies (stage 39, 10 days) [58], and the whole body of medaka were used. In this study we also obtained life span data from hatching to 1 year from part of a population bred in 2009 including the period that had been previously unexamined [38].

The body size data (Table 1) were obtained by sampling for body length and weight from the embryo stage to 4-year-old fish. We measured body length (total body length) (diameter in the case of embryos) to the nearest 0.1 mm using a vernier caliper and body weight to the nearest 1 mg using an analytical balance. Before measurement, the fish were anesthetized using 2-phenoxyethanol solution. We also examined development-related changes in external morphology during the experiment. All procedures were approved by the Nippon Veterinary and Life Science University Research Ethics Committee.

### Analysis of telomere length using Southern blotting

Total DNA was extracted from embryos (stage 39, 10 days, hatching stage) (10 bodies constituting one sample) and the whole body (one individual per sample) (Table 1). Prior to restriction digestion, genomic DNA from all samples was tested for autolytic changes by gel electrophoresis. We used samples that were larger than the 23.1-kbp marker employed in our procedure and appeared as single compact crowns that migrated in parallel with an undegraded intact sample [59]. The terminal restriction fragment (TRF) length derived from *Hin*f I-digested DNA was measured by the standard Southern blotting method described previously [45, 60]. In this study, we used the Telometric software package version 1.2 (Fox Case Cancer Center, USA) to assess the sizes and distribution of TRF [47]. We adopted the median value of the TRF as a representative of telomere length, because the TRF values did not show a Gaussian distribution [38].

Because of criticism that has been directed toward the use of Telometric [26], we re-evaluated the TRF values using ImageJ software version 1.39 (National Institutes of Health, USA). We adopted the mean value of the TRF as a representative of telomere length, because the most frequently used formula is mean TRF = ∑ (ODi x MWi) / ∑ ODi where ODi is the optical density at position i and MWi is the molecular weight at position i [61]. We compared the results of our analysis of Telometric-derived data with those based on data generated by ImageJ.

### Telomerase assays using the Telomeric Repeat Amplification Protocol (TRAP)

Telomerase assays were performed by the standard Telomeric Repeat Amplification Protocol (TRAP) [62]. Briefly, lysates were prepared by powdering samples (Table 1) frozen in liquid nitrogen, followed by homogenization in 200 μl of ice-cold lysis buffer and incubation for 30 min on ice. Assay tubes were prepared by sequestering 0.1 μg of CX primer (5′-CCCTTACCCTTACCCTTACCTAA-3′) (Nihon Gene Research Laboratories Inc., Japan). The extracts, equivalent to 6 μg protein, were assayed in 50 μl of reaction mixture containing TS primer (5′-AATCCGTCGAGCAGAGTT-3′) (Nihon Gene Research Laboratories Inc., Japan), 1 μg of T4 gene 32 protein (Roche, Switzerland), and 2 units of Taq DNA polymerase (Takara Bio Inc., Japan). After 30 min of incubation at room temperature for telomerase-mediated extension of the TS primer, the reaction mixture was heated at 90°C for 90 s and then subjected to 31 polymerase chain reaction (PCR) cycles. The PCR product was then electrophoresed, and the gels were stained with SYBR Green I (Lonza, Switzerland) for 30 min.

We used ImageJ software version 1.39 to quantify the band densities. Relative values of telomerase activity in medaka samples were obtained by calibration with reference values from a human cancer cell line (SiHa cells, positive control). The value of the positive control was defined as ‘one’ [38].

### Statistical analysis

Body size (body length and weight) was analyzed using R software version 3.1.2 (The R Foundation for Statistical Computing) [63]. We applied four models (linear regression, exponential regression, asymptotic regression, and the Gompertz growth model), and selected for the lowest AIC [40, 64]. RSS [40] was used as a reference. We used SAS software version 9.3 (SAS Institute Inc., USA) [65] for telomere (TRF) length and telomerase analysis. The one between linear and nonparametric regression model (LOWESS) [66, 67] for choosing the smoothing parameter value to minimize AIC_C1_ was selected for the lower AIC_C1_ [40, 41], which is the bias-corrected AIC. RSS was given for reference. SAS software version 9.3 was also used for assessing the correlation between telomere (TRF) length and telomerase activity.

## SUPPLEMENTAL FIGURE


