# Supplementary material for: Telomere attrition and restoration in the normal teleost Oryzias latipes are linked to growth rate and telomerase activity at each life stage
Source: Aging (Albany NY). 2016 Jan 20;8(1):62–75. doi: 10.18632/aging.100873 (PMC4761714; doi:10.18632/aging.100873)
Supplement: Supplementary file 1 [file aging-08-062-s001.pdf]

SUPPLEMENTARY FIGURE

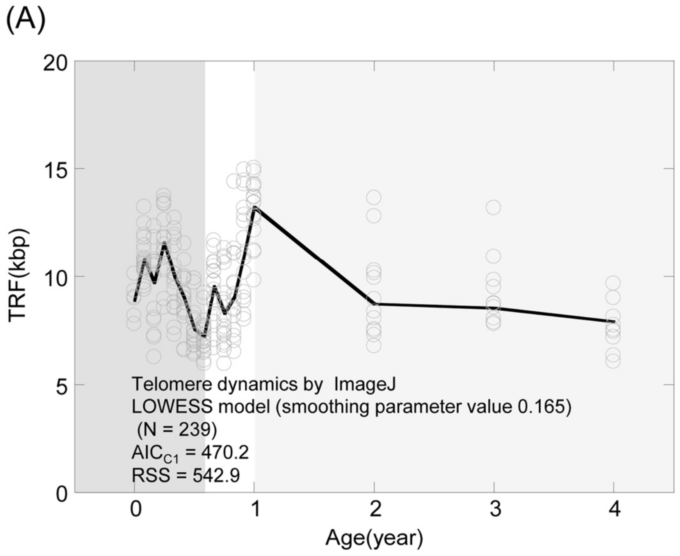

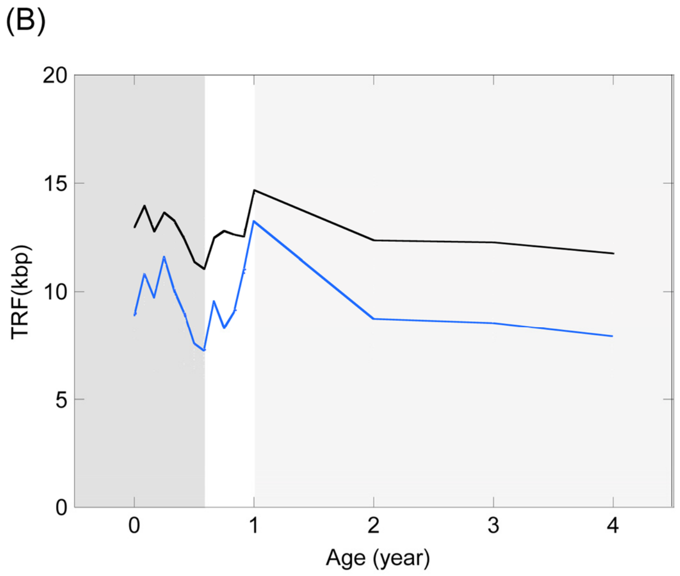

Figure S1. TRF length from embryo to extreme old age in the medaka. (**A**) Scatterplots of telomere dynamics obtained using ImageJ and the fitted regression model. (**B**) Comparison of the regression curve of telomere dynamics obtained using Telometric and ImageJ. Black curve, Telometric; Blue curve, ImageJ. G (dark gray shading), growth stage; Ado (clear), adolescent stage; Adu (light gray shading), adult stage.
